# Re-Isolating *Batrachochytrium dendrobatidis* from an Amphibian Host Increases Pathogenicity in a Subsequent Exposure

**DOI:** 10.1371/journal.pone.0061260

**Published:** 2013-05-06

**Authors:** Forrest M. R. Brem, Matthew J. Parris, Gretchen E. Padgett-Flohr

**Affiliations:** 1 University of Memphis, Department of Biological Sciences, Memphis, Tennessee, United States of America; 2 Southern Illinois University Carbondale, Department of Zoology, Carbondale, Illinois, United States of America; Smithsonian's National Zoological Park, United States of America

## Abstract

Controlled exposure experiments can be very informative, however, they are based on the assumption that pathogens maintained on artificial media under long-term storage retain the infective and pathogenic properties of the reproducing pathogen as it occurs in a host. We observed that JEL284, an *in vitro* cultured and maintained isolate of *Batrachochytrium dendrobatidis* (*Bd*), was becoming less infectious with successive uses. We hypothesized that passing an isolate propagated on artificial media through an amphibian host would make the isolate more infectious and pathogenic in subsequent exposures. To test our hypothesis, we used two discreet steps, a reisolation step (step 1) and a comparative exposure step (step 2). In step 1, we exposed eastern spadefoot toads, *Scaphiopus holbrooki*, to JEL284 and JEL197, another isolate that had been maintained *in vitro* for over six years. We then re-isolated JEL284 only from a successful infection and named this new isolate JEL284^FMBa^. JEL197 did not infect any amphibians and, thus, did not proceed to step 2. In step 2, we compared infectivity and pathogenicity (mortality and survival time) of JEL284 and JEL284^FMBa^ by exposing 54 naïve *S. holbrooki* to three treatments (JEL284, JEL284^FMBa^, and negative control) with 18 individuals per group. We found that JEL284^FMBa^ caused higher mortality and decreased survival time in infected individuals when compared to JEL284 and negative controls. Thus, our data show that pathogenicity of *Bd* can decrease when cultured successively in media only and can be partially restored by passage through an amphibian host. Therefore, we have demonstrated that pathogenicity shifts can occur rapidly in this pathogen. Given the potential for shifts in pathogenicity demonstrated here, we suspect *Bd* to have similar potential in natural populations. We suggest that, when possible, the use of freshly isolated or cryopreserved *Bd* would improve the quality of controlled exposure experiments using this pathogen.

## Introduction

Experimental disease ecology has relied heavily on controlled laboratory experiments when investigating the biology of host-pathogen systems. Controlled exposure experiments have been used to assess modes of transmission and determinants of pathogenicity in many disease systems and have yielded information vital to the management of wildlife diseases. We use controlled pathogen exposure experiments because they are powerful tools that enable manipulation of the three components of host-pathogen systems; host, pathogen, and environment [1], [2]. Controlled exposure experiments have been extensively used to examine the relationship between *Batrachochytrium dendrobatidis* (*Bd*), the etiological agent causing the disease amphibian chytridiomycosis, and its amphibian hosts. Such experiments have been used to investigate determinants of infectivity (ability to infect) and pathogenicity (ability to cause disease given infection) of *Bd* [3], [4], [5], and were also effectively used to demonstrate how *Bd* kills amphibians [6]. Controlled exposure experiments have also proven useful for investigations of potential interactions between amphibian species and *Bd* isolates [7], [8], [9]. However, all such studies are inherently based on the assumption that laboratory host-pathogen models accurately reflect natural host-pathogen interactions, and that pathogens raised strictly in culture retain the same infectivity and pathogenicity as those that reproduce in a host. These assumptions need to be tested, because loss of infectivity and/or pathogenicity has been documented in some fungal pathogens maintained *in vitro* outside their natural host(s) [10].

The source of *Bd* for most exposure experiments is *in vitro* cultures. When using cultured *Bd*, we assume that interactions using these cultures are representative of natural exposures. Specifically, we assume that infectivity and pathogenicity of wild *Bd* and cultured *Bd* are equivalent. In recent years, researchers have demonstrated that there is genetic variability among *Bd* isolates [11], [12], [13] and that infectivity and pathogenicity vary among isolates [14], [7], [8], [9]. Thus, we suspect that genetic variability may explain differences in infectivity and pathogenicity among isolates of *Bd*. However, because pathogens generally adapt rapidly to novel conditions, such as artificial culture, we suggest that it is also important to examine how *in vitro* culture affects infectivity and pathogenicity of *Bd*

In addition to genetic differences among *Bd* isolates, phenotypic differences among isolates may affect infectivity and pathogenicity of *Bd*. *Bd* isolates differ in zoospore production rates *in vitro* [15], a trait that may influence infectivity and pathogenicity of *Bd*. Furthermore, infectivity and pathogenicity of *Bd* can vary among amphibian hosts [7], [9]. These studies are strongly suggestive that there are differences in infectivity and pathogenicity among *Bd* isolates, but whether these differences are genetically or environmentally influenced is unclear. Thus, it is imperative to understand where these variations arose (*in vivo* or *in vitro*), what selection pressures lead to variability among isolates, and, ultimately, how these variations could impact controlled laboratory experiments as well as the management of *Bd* in wild and captive amphibian populations.

Pathogens evolve rapidly in nature [16]. Pathogens in culture can also evolve rapidly through artificial selection on culture media outside their hosts [10]. Pathogens rapidly adapt to novel conditions because, in general, they often have large distribution ranges that are characterized by geographic isolation, large population sizes, short generation times, and high mutation rates. *Bd* has specifically been demonstrated to have high genetic/allelic diversity within populations [17]. *Bd* reproduces primarily asexually and thus, new alleles arising from mutations can easily become fixed in a population, because linkages among genes are not broken by the recombination that occurs in sexual reproduction [18]. Previous research on entomogeneous fungi used for biological control has shown that they attenuate (undergo morphological and physiological change) rapidly in artificial culture [10]. Attenuation can be caused by artificial selection for growth in culture and attenuated pathogens generally suffer from reduced infectivity, reduced pathogenicity, and morphological degeneration if not routinely passed through an appropriate host [10]. It is unclear whether *Bd* attenuates in culture and, if so, how long it takes before infectivity and/or pathogenicity begin to diminish. It is possible that artificial selection could result in attenuation which could underestimate the infectivity and/or pathogenicity of *Bd* in experimental challenges.

Attenuation of *Bd* in culture has not been previously investigated; however, the use of attenuated *Bd* could reduce the accuracy of extrapolating exposure experiments to natural amphibian-*Bd* interactions. We received *Bd* isolate JEL284 in 2007 and JEL 197 in 2008, maintained them in 1% tryptone broth, and passed them to fresh media every 30–45 days. In 2009–2010, we observed that *Bd* isolate JEL284 began to show decreasing infectivity in susceptible amphibian species in controlled exposures. For example, using similar exposure methodologies, JEL284 was highly infectious to adult *Anaxyrus fowleri* and *Hyla chrysoscelis* in 2004 [19] and *Lithobates sphenocephalus* in 2006 (Parris unpublished data). We received freshly thawed JEL284 again in 2007 and it successfully infected larvae of the same species above when used between 2007–2008. We then maintained this isolate *in vitro* as described below. In 2009–2010 we used the *in vitro* maintained JEL284 that we received in 2007 in attempts to expose adults of the above species. However, the exposures to JEL284 performed between 2009–2010 resulted in very low to zero infectivity despite repeated exposures and higher dosages (F. Brem, unpublished data).

The objective of our study was to investigate whether cultured *Bd* isolates may exhibit decreases in infectivity and pathogenicity, and if those decreases could be reversed by passing potentially attenuated *Bd* isolates through an amphibian host. Given the observations concerning the potential attenuation of JEL284 described above, we suspected that conditions associated with propagation on artificial media, such as lower temperatures and the artificial media itself, may be reducing the infectivity and pathogenicity of *Bd* cultures used in controlled exposure experiments. Thus, we hypothesized that passing an isolate propagated on artificial media through an amphibian host would make the isolate more infectious and pathogenic on subsequent exposures. We were unaware of the infectious and pathogenic properties of JEL197. However, like JEL284, our isolate of JEL197 had been maintained *in vitro* under similar conditions for many generations; thus, it may show similar reductions in infectivity and pathogenicity.

## Methods

### Ethics Statement

The experiments herein comply with the current laws of the USA. Collections of *S. holbrooki* were obtained by permits from the Tennessee Wildlife Resources Agency (permit # 3054) and Tennessee Department of the Environment and Conservation (2007-001 and annual renewals). The use of vertebrates in our experiment was approved by the University of Memphis International Animal Care and Use Committee (permit #'s 0650 and 0691).

### Collection and Care of Animals

We collected eastern spadefoot toad, *Scaphiopus holbrooki*, eggs on March 26, 2010 after heavy spring rains at the Edward J. Meeman Biological Field Station approximately 25 miles north of Memphis, TN (35°23′22.66″N 90°02′15.75″W). We used *S. holbrooki* because it is an explosively breeding, fossorial species with high seasonal abundance, it shares breeding site with other local species, and we know little about susceptibility in this species. Explosive breeders are temporally extremely abundant and, if susceptible to *Bd* infection, they could be important ephemeral reservoirs or vectors of *Bd* to other species breeding at the same time or those species with larvae present during *S. holbrooki* breeding events. In a temperature controlled indoor laboratory, the eggs hatched 3–5 days after collection and tadpoles were reared collectively in a 100 L plastic tub at 15–20°C on a 12∶12 light∶dark photoperiod. We performed 50% water changes every 4–5 days, using tap water that was aged in 180 L Nalgene carboys for at least 14 days before use. We changed the water by siphoning approximately 50 L of the existing water and replacing it with 50 L fresh water. We did not do 100% water changes because *S. holbrooki* tadpoles are voracious feeders and, thus, rapidly produces waste. 100% water changes would have been too stark of a change in water chemistry for the tadpoles to adjust to without ill effects. We fed tadpoles *ad lib* daily using ground Hikari Staple™ (Kyorin co., ltd.) koi fish food between the hours of 0800–1700.

All individuals used for the experiment metamorphosed between April 6–13, 2010. After both forelimbs emerged, metamorphs were immediately transferred to individual 0.95 L, polyethylene cups with vented lids, 2.5 cm of moistened sphagnum moss, and a small cutting of Wandering Jew (*Tradescantia zebrine*). We replaced individual containers every two weeks throughout the duration of the experiments. The metamorphs were fed *ad lib* 3 times weekly with a size-appropriate mix of fruit flies (*Drosophila melanogaster* and *D. hydei*), and European house crickets (*Achaeta domestica*), until the start of the experiment. During the experiments, all amphibians were fed size-appropriate *A. domestica* every third day at a rate equivalent to 0.1 g crickets per 1.0 g amphibian mass per day (e.g., a 1 g frog was fed 0.3 g crickets per feeding). Feeding rates for the experiments were determined by average mass at the start of the experiment. Ambient temperature was ∼18°C (range 17–20°C), relative humidity was ∼85% (range 80–100%), and we used a 12∶12 light∶dark photoperiod throughout the experiment.

### Isolates

#### JEL284

This isolate was provided by Joyce E. Longcore (JEL, University of Maine, Bangor, Maine USA) whose source for the pathogen was an infected northern leopard frog, *Lithobates pipiens*, wild-collected from Wisconsin, USA [17] that was received in a shipment from a biological supply house in 2003. We received the isolate in 2007, and prior to use in these experiments we maintained the isolate at 5°C in tryptone liquid media (10 g tryptone, 100 mL deionized water) with 250 mg/L penicillin 300 mg/L streptomycin. We passed colonies monthly to fresh media to reduce potential competition for declining nutrient levels. We used this isolate in both steps 1 and 2 described below.

#### JEL197

In June of 2008, Dr. Michael Levy (North Carolina State University, Raleigh, NC) provided us with JEL 197, the type isolate of *Bd*, for making positive controls for PCR assays. The isolate originally was provided to Dr. Levy by JEL in 2004. We maintained this isolate as described above. JEL197 had been in artificial culture for at least six years and was maintained in conditions similar to JEL284 above; thus, we included it in our design to compare results between two isolates that have been propagated on artificial media under refrigeration.

#### JEL284^FMBa^

In May 2010 (step 1 below), we exposed *S. holbrooki* metamorphs to JEL284 and JEL197 with 20 animals in each treatment group. From this exposure, we successfully infected four individuals (all from JEL284), and then successfully re-isolated *Bd* from one of those infected individuals. This new isolate was renamed JEL284^FMBa^.

### Culture of Isolates

After we re-isolated JEL284^FMBa^, we used antibiotic-free tryptone agar (10 g tryptone, 10 g agar, 1 L deionized water) and tryptone broth (10 g tryptone, 1 L deionized water) for all of our *Bd* cultures. We stopped using antibiotics because we had difficulties isolating *Bd* on agar containing them. All cultures were passed one time before conducting the attenuation test described below. To prepare an inoculate for exposures in steps 1 and 2 described below, we plated 1 mL of active broth onto 25 tryptone agar plates per isolate (10 g tryptone, 10 g agar, 1000 mL deionized water) before harvesting zoospores. To harvest zoospores for exposures, we flooded *Bd* culture plates with 3 mL sterile deionized water for 30 minutes. We then collected the water in a common container prior to estimating zoospore density using a hemacytometer by counting motile zoospores [20]. Before exposure in step 2 described below, we adjusted the zoospore solutions using 17 mL sterile deionized water to JEL284^FMBa^, so that all treatment groups for all experiments were exposed to a concentration of ∼330,000 zoospores/mL. We acknowledge that adding the additional deionized water, instead of water washed over media prior to addition, altered the concentration of media in the inoculate solution and we cannot account for this potentially confounding factor. We chose a relatively high concentration of *Bd* zoospores because we were unsure of the susceptibility of *S. hokbrooki* to *Bd* and wanted to ensure infection given that this species was susceptible to infection, also explosive breeders could be exposed to very high *Bd* levels in nature. Finally we observed motile zoospores in the inoculates using light microscopy before exposing, and after removing, the frogs, thus, we can confidently state that the frogs were exposed to viable *Bd* inoculates.

### Approach

To conduct this experiment, we used two discreet steps, a reisolation step (step 1) and a comparative exposure step (step 2). In step 1, we exposed *S. holbrooki* juveniles to two cultured *Bd* isolates JEL284 and JEL197 that we suspected of reduced infectivity and pathogenicity. We then re-isolated *Bd* (hereafter *Bd* isolate JEL284^FMBa^) from a successful infection caused by JEL284 when thalli were apparent in skin slough. In step 2, we compared pathogenicity of JEL284^FMBa^ and JEL284 in the same host species via exposures to naïve hosts from the same cohort as those used in step 1.

#### Step 1-Exposure and Reisolation of Cultured Bd Isolates

To estimate initial infectivity and pathogenicity of JEL284 and JEL197, and to provide an infected frog from which to re-isolate *Bd*, we randomly assigned 60 of the recently metamorphosed *S. holbrooki* froglets to be used in step one. We then randomly assigned those 60 frogs to one of three treatment groups, JEL284, JEL197, and negative control. At the time of exposure, all metamorphs appeared healthy, were between 10–14 days post-metamorphosis, and weighed between 0.95 and 1.25 g. We did not swab frogs for PCR testing prior to the study because they were collected as eggs, raised in aged tap water, and we assumed that they should be negative. We did, however, collect a skin scraping from 50% of these frogs prior to the start of this experiment and found no evidence of *Bd* thalli, and, thus, we assumed they were *Bd* negative. Frogs in each treatment group were individually exposed to 3 ml of either JEL284 or JEL197 in a 45 mm petri dish for 12 hours. We weighted the lids with small rocks to prevent animals from escaping. To control for the effects of media in the inoculates on amphibian survival, we exposed frogs in the negative control group to 3 mL of an equal parts mixture of heat-killed JEL284 and JEL197 as described above. We mixed the heat-killed isolates together for the negative control to account for possible variation in metabolic products of the isolates. We heat-killed *Bd* immediately after the live exposures by heating the *Bd* inoculates in a warming oven at 75°C for one hour, after which no motile zoospores could be observed. After day 30, all remaining individuals were euthanized in a 500 mg/L solution of MS-222.

After exposing the frogs to the *Bd* treatments, we placed them into freshly prepared containers as described above, and randomly assigned them a position in our experimental area. We observed individuals daily for changes in behavior suggesting clinical symptoms of *Bd* (lethargy, lack of burrowing, and reduction in feeding), morbidity, and mortality for 30 days post-exposure. To determine post-exposure infection status, we repeated the above light microscopy assay at day 14 when we began to observe clinical symptoms of amphibian chytridiomycosis in some individuals. We again repeated the light microscopy assay at day 30 when the individuals exhibiting clinical symptoms had died and no other frogs were exhibiting similar symptoms. After we confirmed infection in any individuals, we conducted daily skin scrapings to obtain epithelial tissue with *Bd* thalli present which was used to re-isolate *Bd* for use in step 2, described below. We only observed tissue with *Bd* thalli and successfully re-isolated *Bd* from JEL 284, thus, JEL 197 was not used in step 2.

#### Step 2-Comparative Exposure Between Cultured and Re-isolated Bd

To test the hypothesis that re-isolated *Bd* would exhibit increased infectivity and pathogenicity, we randomly assigned 54 naive *S. holbrooki* juveniles from the same cohort to one of three treatment groups: JEL284, JEL284^FMBa^, and negative control with 18 replicates per treatment. These frogs were of the same cohort in step 1, but were older (between 60–75 days post-metamorphosis) and larger (between 1.85–2.5 g) as our approach prohibited us from conducting the two steps concurrently. No frogs from step 1 were used in step 2.

We exposed frogs and monitored them post-exposure using the methods described in step 1 above with only a few exceptions. The negative control we used for step 2 was a mixture of heat-killed JEL284 and JEL284^FMBa^. In step 2, we monitored frogs for 60 days to ensure ample time for infections to progress to disease. We recorded dates of all mortality events for survival analysis and interpreted survival time as time from exposure to death. At two weeks post-exposure, we swabbed all frogs to determine infection status. We sent the swabs to Pisces Molecular, LLC. for conventional PCR assay.

### Statistical Analyses

We tested for differences in *Bd* infectivity and host mortality between the treatments using chi-square tests. We tested for differences in survival times using Kaplan-Meier log-rank survival analysis.

## Results

### Step 1 – Exposure and Reisolation of Cultured Bd Isolates

Using light microscopy of skin scrapings we found that JEL284 infected 4 of 20 (0.20, 95% CL 0.06–0.44) and both JEL197 and the negative control infected 0 of 20 (0.00, 95% CL 0.00–0.17). All four infections identified in the JEL284 group were evident by light microscopy at day 14 when we began re-isolation attempts. In addition, all four individuals we diagnosed as infected eventually died of amphibian chytridiomycosis at days 22, 24 (2), and 27. However, before they died, we successfully re-isolated *Bd* from one of four moribund individuals infected with JEL284 for use in step 2. The individual from which we isolated JEL284^FMBa^ was one that died at day 24. We diagnosed amphibian chytridiomycosis as the cause of death because these animals: stopped eating, were lethargic, ceased burrowing into the substrate, developed seizure like episodes days before death, had nearly fully coalescent coverage of *Bd* thalli in the epidermis, and the lack of all such symptoms in the negative control frogs and other uninfected individuals.

### Step 2 – Comparative Exposure Between Cultured and Re-isolated Bd

#### Mortality

**Figure 1:**
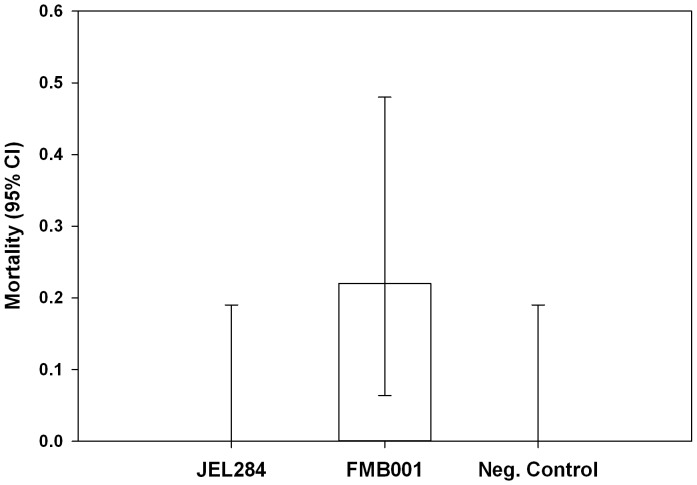
Virulence of two *Batrachochytrium dendrobatidis* (*Bd*) isolates to *Scaphiopus holbrooki* with reference to a negative control (exposed to heat-killed *Bd*) measured by mortality. Mortality of frogs to JEL284 was similar to negative controls, whereas mortality in frogs exposed to JEL284^FMBa^ was significantly higher than both JEL284 and negative controls.

JEL284 infected 3 of 18 *S. holbrooki* (0.17, 95% CL0.04–0.41), JEL284^FMBa^ infected 7 of 18 (0.39, 95% CL 0.17–0.64), and the negative control (NC) infected 0 of 18 (0.00, 95% CL 0.00–0.19) individuals. Four of the 18 *S. holbrooki* that were exposed to JEL284^FMBa^ were diagnosed with *Bd* infection and subsequently died of amphibian chytridiomycosis at days 20, 25, 26, and 29 (Figure 1). Only frogs that died showed overt signs of amphibian chytridiomycosis, all other individuals appeared healthy. We found overall significant differences in mortality among *Bd* treatment groups, χ^2^ (df = 2, n = 54) = 8.64, *p* = 0.0133. *Post hoc* analysis indicated that there was a significant difference in pathogenicity between JEL284^FMBa^ and JEL284 χ^2^ (df = 1, N = 36) = 4.50, *p* = 0.034. Thus, JEL284^FMBa^ caused higher mortality and was, therefore, more pathogenic than JEL284 in *S. holbrooki* (Figure 1).

#### Survival Time

**Figure 2:**
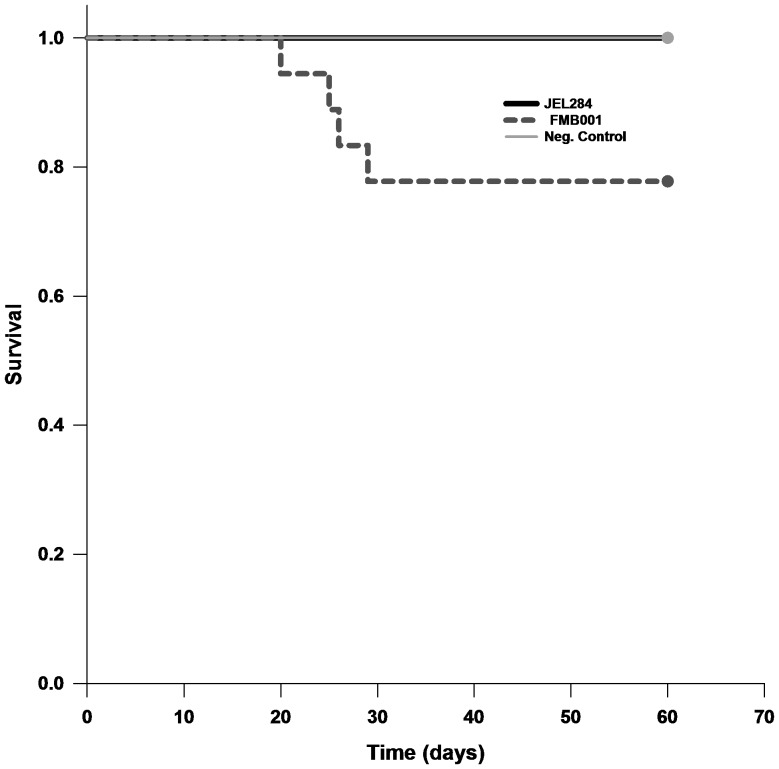
Survival time of *Scaphiopus holbrooki* after exposure to two *Batrachochytrium dendrobatidis* (*Bd*) isolates with reference to a negative control (exposed to heat-killed *Bd*) as measured by time to death. Time to death of frogs exposed to JEL284 was similar to negative controls, whereas time to death in frogs exposed to JEL284^FMBa^ was significantly higher than both JEL284 and negative controls.

Kaplan-Meier survival analysis (Figure 2) revealed an overall significant difference in survival times among frogs exposed to different *Bd* isolates (log-rank test, z = 8.749 df = 2 p = 0.013). A *post hoc* test directly comparing JEL284 and JEL284^FMBa^ indicated there was a difference in survival time between frogs exposed to these *Bd* isolates (Holm-Sidak Test p = 0.036). Thus, frogs exposed JEL284 lived longer than those exposed to JEL284^FMBa^.

## Discussion

Our work shows that passing long-term cultured *Bd* through an amphibian host can increase the pathogenicity of the isolate in a subsequent exposure (Figures 1 and 2). In step 2, no frogs in the JEL284 group died or showed any clinical symptoms of amphibian chytridiomycosis during the 60-day study period. Conversely, four frogs infected with JEL284^FMBa^ died of chytridiomycosis before day 60 and survival time of frogs exposed to JEL284^FMBa^ was significantly decreased. Thus, JEL284^FMBa^ was more pathogenic to *S. holbrooki* than JEL284 (Figures 1 and 2). Our results suggest that pathogenicity of *Bd* can shift rapidly when subjected to novel conditions. Thus, we speculate that pathogenicity of cultured *Bd* may not necessarily be equivalent to pathogenicity of wild *Bd*, and we suggest future research should examine the effects of factors related to maintenance on media (i.e. growth outside natural host, low temperatures, and antibiotics) over time on *Bd* infectivity and pathogenicity.

To approximate natural pathogenicity in controlled exposure studies, *Bd* used in such studies should be recently isolated from wild amphibians when possible. However, we acknowledge that isolating *Bd* is a difficult task that is not always successful so using freshly isolated *Bd* will not always be possible. Alternatively, revived cryopreserved isolates could be used if they were cryopreserved soon after isolation from an amphibian host. However, we do not know whether the cryopreservation process imposes artificial selection. Artificial selection could introduce genetic variability not representative of wild *Bd*, thus, we suggest that freshly isolated *Bd* should be used when possible. For controlled experiments to most accurately reflect natural host-pathogen interactions and make meaningful contributions to disease ecology, we must minimize potential sources of artificial selection before exposing hosts to cultured *Bd*.

When using cultured pathogens, many steps in the isolation and culture of a pathogen may impose artificial selection that could impact experimental results. Mutations in culture could promote a tradeoff where parasites become efficient at converting artificial media to new parasite production, and less efficient at doing so using host tissues [10]. In another study, the reverse was demonstrated where an opportunistic pathogen showed decreased saprobic capacity after serial passage through a host [21]. Knowledge of the reproductive system of *Bd* is incomplete, but work suggests that *Bd* may be vegetatively diploid, and possibly asexual [22]. Thus, mutations beneficial for growth in culture would be expected to be rapidly fixed in an asexual organism due to the lack of recombination that allows associations between loci to be maintained [18]. We also suggest that the use of antibiotics in *Bd* media may be a possible source of artificial selection leading to attenuation for growth on media. There is evidence of negative effects of antibiotic use in fungal cultures in the *Bd* and other systems [23], [24], [25] and antibiotic use is commonplace in maintaining *Bd* on media [23], [26]. Finally, temperature has been shown to affect reproductive life history patterns of *Bd* [27]. *Bd* is generally kept at a temperature only a few degrees above freezing in culture. Researchers later expose amphibian hosts to potentially cold-adapted *Bd* at higher temperatures. Fungi are well known for adapting to cold climates [28], [29]; thus, directional selection for enhanced growth in colder conditions is possible. If *Bd* is undergoing selection for enhanced growth on media or at low temperatures, we suggest a tradeoff likely exists where *Bd* may become less efficient at utilizing host tissues for reproduction and virulence in the host could decrease as a result.

We have demonstrated that change in *Bd* can occur within the course of a single infection in an amphibian host. The original *S. holbrooki* individual infected with JEL284, from which we isolated JEL284^FMBa^, was infected for just under one month before it began to show clinical symptoms of amphibian chytridiomycosis, became moribund, and eventually died at day 24. These results indicate that shifts in the pathogenicity of *Bd* can occur very rapidly and within the infection of a single host. Recent evidence has shown that distinct lineages of *Bd* have arisen in nature [11], [30] and that some of these lineages are more pathogenic than others [11]. Identifying genetic changes that affect the pathogenicity of *Bd* is critical for understanding factors that determine the outcomes of *Bd*-amphibian interactions. Genomic studies have also identified potential genes involved in *Bd* pathogenicity [31]. We suggest that future research should determine if pathogenicity shifts, such as those we have demonstrated, have a genetic basis (i.e. artificial selection and attenuation) or are the result of phenotypic plasticity.

Although we have shown that *Bd* can demonstrate rapid changes in pathogenicity, our data should be interpreted cautiously. *Scaphiopus holbrooki* seems to have low susceptibility to *Bd* infection as was shown in this exposure experiment and a recent field survey [32]. However, if the experiment were conducted with a highly susceptible species, results were likely to have been more dramatic. We chose *S. holbrooki* because we wanted to determine susceptibility in this species because it is an explosive breeder that overlaps with both late winter and spring breeding species. Thus, *S. holbrooki* could be an important reservoir or vector of *Bd*. We did not include a cryopreserved JEL284 as a reference for wild-type properties of this isolate. It is possible that JEL284 naturally exhibits low infectivity. However, JEL284 was highly infectious to adult *Rana pretiosa* [33] and larval *Anaxyrus fowleri* and *Hyla chrysoscelis* [34], [35]. In addition, a controlled exposure study [33] using the same two isolates (JEL284 and JEL284^FMBa^) and a different host, the Oregon spotted frog (*Rana pretiosa*), corroborated our results. *Rana pretiosa* exposed to JEL284^FMBa^ and JEL284 were 100% susceptible to both isolates, however those exposed to JEL284^FMBa^ gained less mass and took longer to clear their infections than those exposed to JEL284. Again, indicating higher pathogenicity of JEL284^FMBa^. Finally, in step 2, we swabbed at day 14 to determine infection status, but not at day 60 at the conclusion of the experiment. We suggest more frequent diagnosis of infection status to determine if individuals had cleared their infections or still carried aclinical infections at the end of the study.

Despite the limitations of the study, our data clearly suggest that *Bd* maintained on media may exhibit shifts in pathogenicity in a relatively short time frame and result in decreased pathogenicity in some amphibian hosts. Our data emphasize that traits influencing the infectivity and pathogenicity of *Bd* can change rapidly. Therefore, the choice as to which amphibian host and *Bd* isolate to use in exposure studies and are important factors that deserve careful consideration. By understanding what makes some pathogen isolates more pathogenic than others we may be able to more effectively manage that pathogen in wild and captive populations. Finally, we suggest that the cultural conditions after isolation of *Bd* isolates used for controlled exposure studies must be considered, and the inference limits of controlled *Bd* exposure experiments should be viewed with caution.
